# Editorial: Machine learning and artificial intelligence for smart agriculture

**DOI:** 10.3389/fpls.2022.1121468

**Published:** 2023-01-09

**Authors:** Chuanlei Zhang, Dong Sun Park, Sook Yoon, Shanwen Zhang

**Affiliations:** ^1^ Artificial Intelligence College, Tianjin University of Science and Technology, Tianjin, China; ^2^ Department of Electronics Engineering, Jeonbuk National University, Jeonju, Republic of Korea; ^3^ Department of Computer Engineering, Muan, Republic of Korea; ^4^ Information Engineering College, Xijing University, Xi’an, China

**Keywords:** computer vision, internet of things, agricultural robot, pest control, food security

## 1 Introduction

Information, knowledge and equipment are the three main components of smart agriculture. This special edition focuses on a few issues that still require research and discussion. For example, using and enhancing machine learning techniques for crop disease and pest detection and recognition, plant species recognition, smart agricultural IoT, food material supply chain security tracing, and other crucial issues in smart agriculture.

## 2 Computer vision

Visual recognition technology has been increasingly applied to numerous areas of agricultural development with the advancement of computer graphics and image processing technology in artificial intelligence (AI). Today, there is still a significant room for this technology in modern agriculture (Tombe, 2020; Benos et al., 2021; Dhanya et al., 2022).

During the process of green apple harvesting or yield estimation, the accurate recognition and fast location of the target fruit bring tremendous challenges to the vision system. Sun et al. improved a density peak cluster segmentation algorithm for RGB images with the help of a gradient field of depth images to locate and recognize target fruit. Specifically, the image depth information is adopted to analyze the gradient field of the target image.

There are two serious apple diseases which are ring rot produced by Botryosphaeria dothidea and anthracnose caused by Colletotrichum gloeosporioides. In Feng et al., the automatic separation between two diseases was examined using image processing technologies. The acquired disease images were preprocessed using morphological opening and closing reconstruction, color image contrast stretching, and image scaling. Then, two crop leaf lesion segmentation algorithms based on circle fitting were suggested and applied. Support vector machine (SVM) models and random forest models were used based on individual LBP histogram features and various LBP histogram feature combinations.

In addition, crop classification is crucial for the development of phenotypes and genetic resources. Fu et al. created rapeseed dataset (RSDS) using eight categories of data gathered. The target-dependent neural architecture search (TD-NAS) was proposed. TD-NAS is a revolutionary target-dependent search technique based on VGGNet.

## 3 Internet of things

Sensor nodes in agricultural IoTs, such as soil temperature and humidity sensors, air temperature and humidity sensors, etc., collect data in the agricultural environment. The data is wireless transferred from the sensor nodes to the sink node for data collection. The gateway change the protocol into one that can be communicated over the Internet when it receives data from the sink node (Ayaz et al., 2019; Doshi et al., 2019; Priya et al., 2021).

Usually, multidimensional time series data are produced in enormous quantities by smart agricultural IoT. However, due to the limitations of the scenarios, data loss and misrepresentation are frequent problems with smart agricultural IoTs. In order to solve the aforementioned issues, in, using generative adversarial networks (GAN), Cheng et al. offered a new anomaly detection model that can handle the multidimensional time series data produced by smart agricultural IoTs.

Meanwhile, a multi-objective strategy based on supervised machine learning was utilized in Uyeh et al. to identify the ideal number of sensors and installation locations in a protected cultivation system. A tree-based model in the form of a gradient boosting technique was specifically adapted to observed (temperature and humidity) and derived circumstances (dew point temperature, humidity ratio, enthalpy, and specific volume). Time series forecasting was used for feature variables. In order to choose the right number and locations for the best sensors in a protected cultivation system, a machine learning model was created and put forth.

In larger fields, such as those seen in large-area agriculture, more sensors/nodes are advised to better account for soil heterogeneity. But farmers must pay a higher and frequently prohibitive price for this (purchase, labor costs from installation and removal, and maintenance). The agricultural industry would benefit greatly from methodologies that allow for maintaining monitoring capability/intensity with fewer in-field sensors. In Maia et al., sensor data analysis over two irrigation seasons in three cotton fields from two cotton-growing regions of Australia revealed a connection between soil matric potential and cumulative crop evapotranspiration (ETcn) derived from satellite measurements between irrigation events. This connection can be represented as a second-degree function.

## 4 Agricultural robots

Globally, the use of intelligent machines and robots in agriculture is receiving increasing attention (Oliveira et al., 2020). A picking robot is a type of agricultural robot that uses a variety of sensors to sense the complicated agricultural environment and then picks the target using this knowledge and a decision-making algorithm. Ma et al. explore the distributed averaging issues of agriculture picking multi-robot systems under directed communication topologies by utilizing the sampled data. A distributed protocol based on nearest-neighbor information is presented using the principles of algebraic graph theory and matrix theory.

## 5 Pest control

Crop diseases and pests have long been a significant issue in agricultural production, having a negative impact on farmers’ income, modern agricultural development, and agricultural output. Early disease and pest identification, monitoring, and control are crucial for preventing the large-scale spread of diseases and pests, preserving the quality of crops, and reducing environmental pollution brought on by pesticide residues (Buja et al., 2021; Liu and Wang, 2021).

The brown planthopper (BPH), Nilaparvata lugens (Stl; Hemiptera: Delphacidae), is a piercing-sucking insect that seriously harms rice plants by sucking out their phloem sap and spreading viruses. For reducing mating rates, a physical control mechanism based on BPH courting disruption is a viable approach to reducing environmental pollution. To gather effective courtship disrupting signals. Feng et al. created a vibration signal recording, monitoring, and playback system for BPHs. This technology was used to gather and evaluate male competitiveness and BPH courting signals in order to determine their frequency spectra. According to the findings, the mean main vibration frequency and mean pulse rate of female courtship signals are 234 Hz and 23 Hz, respectively. Male courting signals had mean main vibration and pulse frequencies of 255 Hz and 82 Hz, respectively.

Furthermore, Cnaphalocrocis medinalis, Sogatella furcifera, and Nilaparvata lugens are three kinds of migratory pests that severely reduce rice yield and result in economic losses each year. Sun et al. create an intelligent monitoring system of migrating pests based on searchlight trap and machine vision to replace manual identification of migratory pests in. The system consists of a cloud server, a Web client, a migratory pest automatic identification model, and a searchlight trap based on machine vision. The searchlight trap uses lights at night to draw in high-altitude migrating insects. All captured insects are distributed using rotary brushes and multi-layer insect conveyor belts. The intelligent monitoring system can automatically monitor the three migratory pests in time.

## 6 Food security

For the quality and safety of agricultural products, food traceability is crucial (Ivar et al., 2020). In Jing and Li a hybrid mode of block-chain and IoTs is used to build a traceability system for red jujube. The solution addresses the issue of date and quality traceability by integrating block-chain and IoTs technologies with properties of tamper-proof, decentralization, and distributed storage. The entire process of red jujube cultivation, processing, and sales is documented in the block. To guarantee the realization of quality traceability of red jujube in the framework throughout the whole process of big data processing, and the crucial data gathered in each procedure is saved in the database.

## Author contributions

The four professors contributed to edit and review the manuscripts for this edition. CZ was mainly responsible for the computer vision and food security part, DP was responsible for the Iot and robot, SY was responsible for the pest control, and SZ was responsible for the Iot and computer vision part. All authors contributed to the article and approved the submitted version.
